# Supplementary material for: Distribution and diversity of dimetal-carboxylate halogenases in cyanobacteria
Source: BMC Genomics. 2021 Aug 31;22:633. doi: 10.1186/s12864-021-07939-x (PMC8406957; doi:10.1186/s12864-021-07939-x)
Supplement: Supplementary file 1 — Additional file 1: Table S1. Accession numbers of cylC homologs and aurFgenes used for primer design. Figure S1. (a) Phylogenetic tree (FastTree GTR with a rate of 100) of cylC homologs highlighted according to the groups selected for degenerate primer design. (b) Schematic representation of the different pairs of degenerate primers. Figure S2. PCR-based detection of cylC homologs in the LEGEcc. Five pairs of primers were designed based on conserved regions identified in the cylC gene. Each primer pair was used in a PCR screen of the gDNA obtained from diverse strains (n = 326) of the LEGEcc. The resulting amplicons were cloned and sequenced. Sequences for each primer pair were aligned with the corresponding regions of cylC genes found in the NCBI reference genomes (cyanobacteria only) and those from LEGEcc strains’ genomes. Shown are the resulting cladograms (RaxML, 1000 replicates) for each primer pair used in the screening. Blue squares indicate sequences obtained from the PCR screen. Figure S3. RaxML cladogram (1000 replicates) of the 16S rRNA gene of LEGEcc strains (grey squares) and from cyanobacterial strains with NCBI-deposited reference genomes, screened in this study. Taxonomy is presented at the order level (colored ranges). Strains whose genomes encode CylC homologs are denoted by black squares. Green squares indicate that at least one CylC homolog was detected by PCR-screening and verified by retrieving the sequence of the corresponding amplicon through cloning followed by Sanger sequencing. The cladogram topology is the same as shown in Fig. 3 of the main manuscript, but here bootstrap values (equal or above 0.7) are shown. Table S2. GenBank or RefSeq assembly acession number and LEGEcc genome used for CORASON analysis. Table S3. BLASTp search of CylC homologs against Aliterella sp., Chroococcidiopsis sp. and Gloeobacter sp. Figure S4. Rieske-containing biosynthetic gene clusters encoding CylC homolog(s). Figure S5. PriA-containing biosynthetic gene cl [file 12864_2021_7939_MOESM1_ESM.pdf]

Supplementary Material

Distribution and diversity of dimetal-carboxylate halogenases in cyanobacteria

Nadia Eusebio[1], Adriana Rego[1], Nathaniel R. Glasser[2], Raquel Castelo-Branco[1], Emily P. Balskus[2,*] and Pedro N. Leão[1]*

[1]Interdisciplinary Centre of Marine and Environmental Research (CIIMAR/CIMAR), University of Porto, Matosinhos, Portugal

[2]Department of Chemistry and Chemical Biology, Harvard University, Cambridge, MA, USA

*Corresponding authors, E-mail: pleao@ciimar.up.pt, balskus@chemistry.harvard.edu

Table S1. Accession number of *cylC* homologs and *aurF* genes used for primer design.

Name	Acession number
Anabaena cylindrica PCC 7122	AP018166.1:4354075-4355373
Anabaena cylindrica PCC 7122	CP003659.1:3493241-3494452
Anabaena minutissima UTEX B 1613	MH325199.1:28248-29645
Aulosira laxa NIES-50	AP018307.1:7246161-7247336
Calothrix brevissima NIES-22	AP018207.1:8432194-8433369
Calothrix parasitica NIES-267	AP018227.1:c657283-656117
Calothrix sp. 336/3	CP011382.1:4155917-4157092
Calothrix sp. NIES-2098	AP018172.1:4832715-4833896
Calothrix sp. NIES-2100	AP018178.1:1190855-1192015
Calothrix sp. NIES-4071	AP018255.1:772595-773998
Calothrix sp. NIES-4071	AP018255.1:7573114-7574265
Calothrix sp. NIES-4105	AP018290.1:7570946-7572097
Calothrix sp. NIES-4105	AP018290.1:c773967-772564
Crinalium epipsammum PCC 9333	CP003620.1:c2520624-2519380
Cyanobacterium aponinum PCC 10605	CP003947.1:1114945-1116162
Cyanothece sp. PCC 7822 plasmid Cy782201	CP002199.1:360048-361412
Cylindrospermum licheniforme UTEX B 2014	KX682397.1:2961-4376
Cylindrospermum sp. NIES-4074	AP018269.1:2484675-2486075
Cylindrospermum stagnale PCC 7417	CP003642.1:c2217555-2216140
Cylindrospermum stagnale PCC 7417	CP003642.1:c2308133-2306952
Cylindrospermum stagnale PCC 7417 plasmid pCYLST.01	CP003643.1:56891-58261
Fischerella sp. NIES-4106	AP018298.1:1441529-1442746
Fremyella diplosiphon NIES-3275	AP018233.1:1201630-1202805
Geitlerinema sp. PCC 7407	CP003591.1:c2644190-2642961
Microcoleus sp. PCC 7113	CP003630.1:c2944932-2943757
Moorea bouillonii PNG5-198	KP715425.1:20564-21952
Moorea bouillonii PNG5-198	KP715425.1:22089-23501
Moorea producens JHB	CP017708.1:c2514441-2513215
Moorea producens PAL-8-15-08-1	CP017599.1:c2474933-2473707
Nodularia sp. HBU26	KY594676.1:18618-19922
Nostoc carneum NIES-2107	AP018180.1:4766806-4767993
Nostoc commune HK-02	AP018326.1:6163477-6164874
Nostoc flagelliforme CCNUN1	CP024785.1:1135915-1137312
Nostoc punctiforme PCC 73102	CP001037.1:4184899-4186269
Nostoc punctiforme PCC 73102,	CP001037.1:c5828161-5826956
Nostoc sp. CAVN2	KT826756.1:3912-5327
Nostoc sp. CCAP 1453/38	KP143720.1:21035-22441
Nostoc sp. CCAP 1453/38	KP143720.1:22490-23818
Nostoc sp. CENA543	CP023278.1:c1270872-1269652
Nostoc sp. '*Lobaria pulmonaria'* (5183)	CP026692.1:3554655-3555860
Nostoc sp. NIES-4103	AP018288.1:c2213285-2211918
Nostoc sp. NIES-4103	AP018288.1:c3294496-3293132
Nostoc sp. PCC 7524	CP003552.1:c5619284-5618085
Nostoc sp. 'Peltigera membranacea cyanobiont' N6	CP026681.1:c3280480-3279275

Nostoc sp. UIC 10110	KY379971.1:817-2223

Table S1. (continued)

Nostocales cyanobacterium HT-58-2	CP019636.1:1098629-1099993
Oscillatoria acuminata PCC 6304	CP003607.1:c70363-69134
Pleurocapsa sp. PCC 7327	CP003590.1:298214-299635
Rivularia sp. PCC 7116	CP003549.1:c7519531-7518356
Stanieria sp. NIES-3757	AP017376.1:c59518-58163
Streptomyces thioluteus HKI-22	AJ575648.1:4858-5868
Synechocystis salina LEGE 06099	KX083339.1:12314-13522
Synechocystis salina LEGE 06155	KR059027.1:13578-14783
Synechocystis sp. IPPAS B-1465	CP028094.1:c2118162-2116924
Synechocystis sp. PCC 6714	CP007542.1:c2270478-2269219
Synechocystis sp. PCC 6803	CP003265.1:c2114589-2113351
Tolypothrix tenuis PCC 7101	AP018248.1:c3613364-3612189

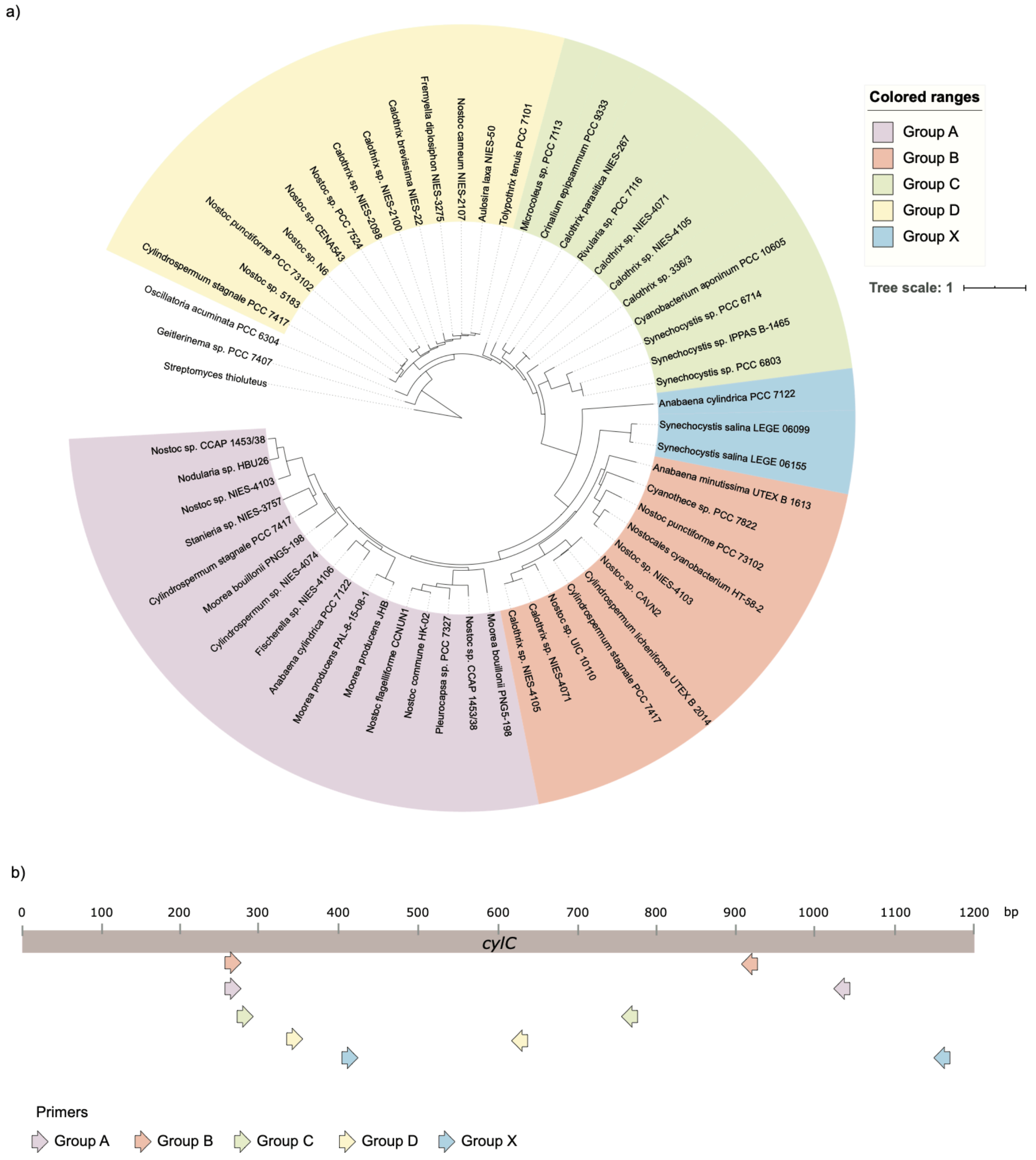

Figure S1. (a) Phylogenetic tree (FastTree GTR with a rate of 100) of *cylC* homologs highlighted according to the groups selected for degenerate primer design. (b) Schematic representation of the different pairs of degenerate primers

Group of primers A

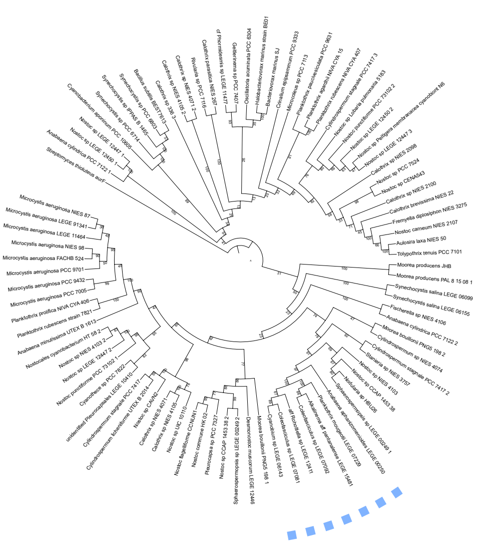

Group of primers B

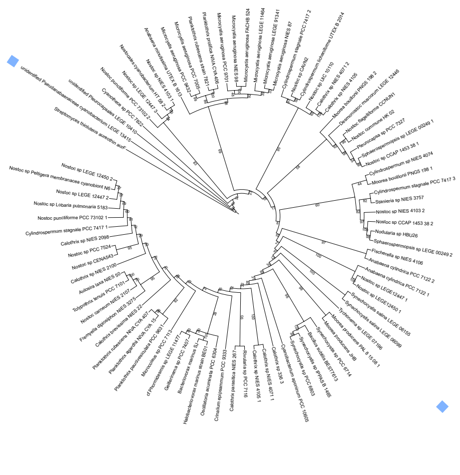

Group of primers C

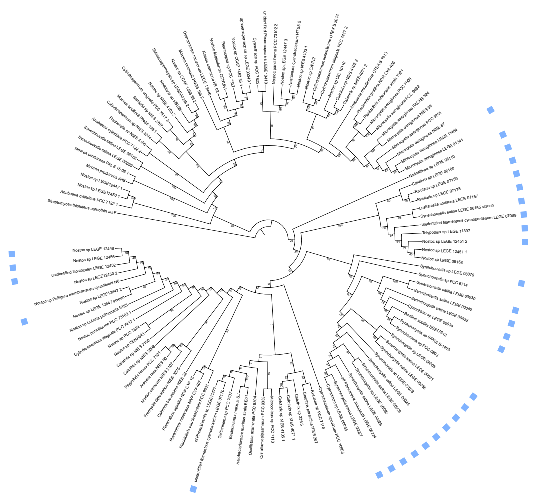

Group of primers D

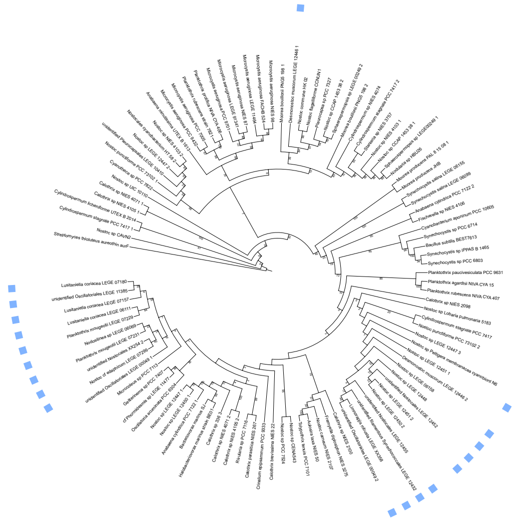

Group of primers X

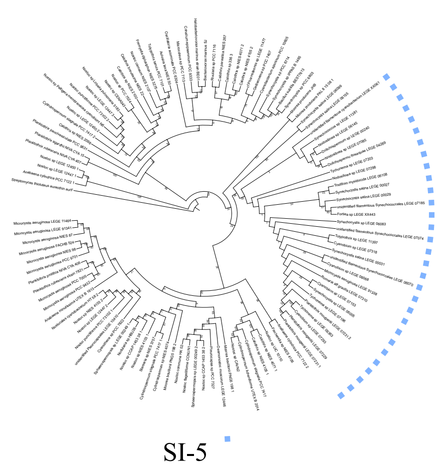

Figure S2. PCR-based detection of *cylC* homologs in the LEGEcc culture collection. Five pairs of primers were designed based on conserved regions identified in the *cylC* gene. Each primer pair was used in a PCR screen of the gDNA obtained from diverse strains (n = 326) of the LEGEcc culture collection. The resulting amplicons were cloned and sequenced. Sequences for each primer pair were aligned with the corresponding regions of *cylC* genes found in the NCBI reference genomes (cyanobacteria only) and those from LEGEcc strains' genomes. Shown are the resulting cladograms (RaxML, 1000 replicates) for each primer pair used in the screening. Blue squares indicate sequences obtained from the PCR screen.

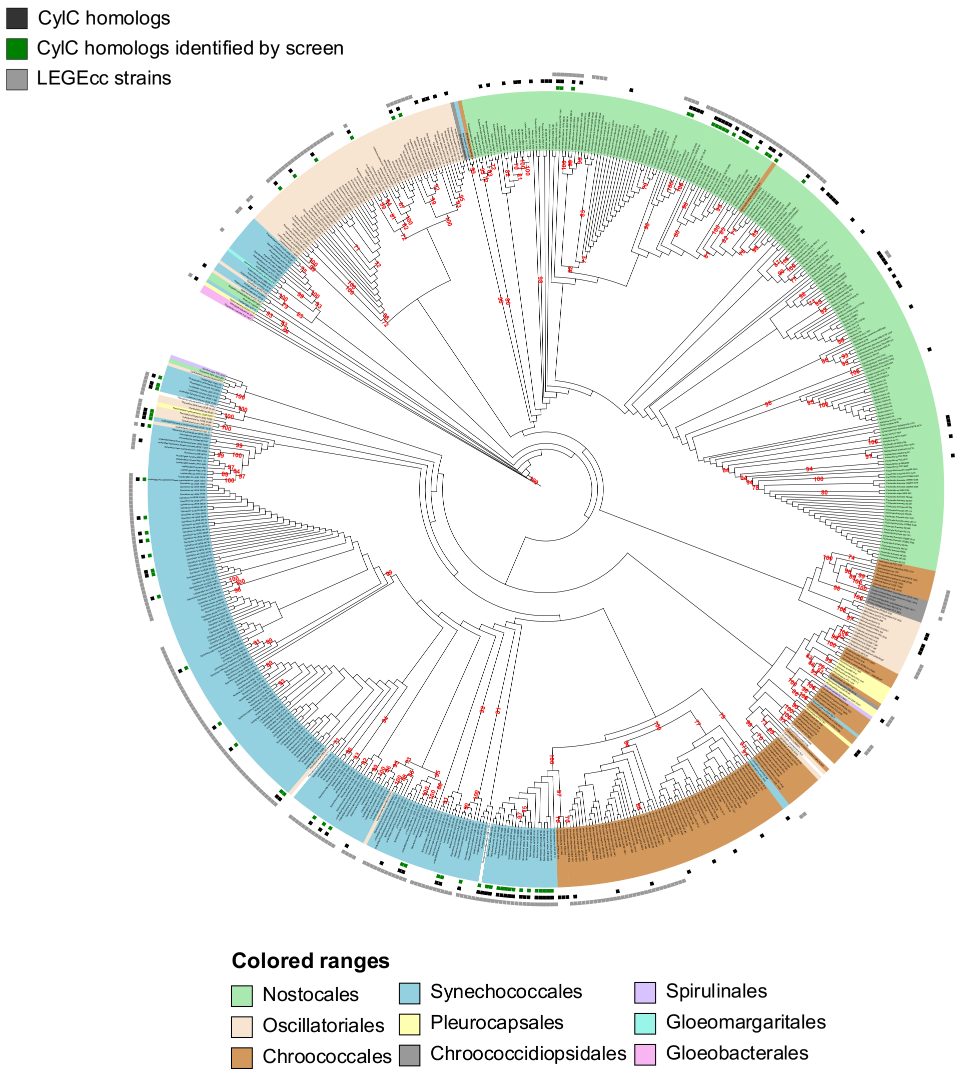

Figure S3. RaxML cladogram (1000 replicates) of the 16S rRNA gene of LEGEcc strains (grey squares) and from cyanobacterial strains with NCBI-deposited reference genomes, screened in this study. Taxonomy is presented at the order level (colored ranges). Strains whose genomes encode CylC homologs are denoted by black squares. Green squares indicate that at least one CylC homolog was detected by PCR-screening and verified by retrieving the sequence of the

corresponding amplicon through cloning followed by Sanger sequencing. The cladogram topology is the same as shown in Figure 3 of the main manuscript, but here bootstrap values (equal or above 0.7) are shown.

Table S2. GenBank or RefSeq assembly acession number and LEGEcc genome used for CORASON analysis.

Enzyme	Reference genome	Number of genomes/Clusters tested in CORASON	Removed from the alignment	Included manually extracted BGCs from genomes
CylC	GCA_000317535.1	2170	GCA_003504865.1, LEGE00239, GCA_000332035.1, GCA_004305995.1, GCA_003206555.1, GCA_000312205.1	CP011382.1, CP026692.1, NZ_LN887838.1, NZ_LN887838.1, KT826756.1, KP143720.1, NZ_CP023280.1, KY594676.1, NZ_CP028099.1, LEGE00239, LEGE_00249, LEGE06071, LEGE06083, LEGE06083, LEGE_06099, LEGE_06155, LEGE07170, LEGE11397, LEGE_11477, LEGE11479, LEGE11480, LEGE12450, LEGE12450, , NZ_CP026681.1, AP018172.1, AP018178.1, AP018180.1, AP018207.1, AP018227.1, AP018233.1, AP017375.1, AP018307.1, NZ_AVFS00000000.1, NZ_AVFV00000000.1, NZ_AVFV00000000.1, NZ_KE734719.1, NC_019776.1", NC_019693.1, CP007542.1, CP003265.1, NZ_AQPY00000000.1, NZ_AP018248.1, CP003630.1, CP003549.1, CP003591.1, CP003642.1, CP003552.1, CP003620.1, NZ_ALVX00000000.1, NZ_ALVX00000000.1, NZ_HE972553.1, NZ_CZCS00000000.2, KY379971.1, MH325199.1, KX682397.1
PrnA	GCA_005518235.1	2114	GCA_002025445.1, GCA_002025445.1	-
CurA	GCA_001942475.1	2116	-	-
mcnD	GCA_000312245.1	2114	LEGE 17501, LEGE 91341	-
hs_bmp5	GCA_001942475.1	2114	GCA_005518205.1, GCA_005518235.1	-
WelO5	GCA_000447295.1	2116	-	-

Table S3. BLASTp search of CylC homologs against *Aliterella sp.*, *Chroococcidiopsis sp.* and *Gloeobacter sp.*

**Input**	**CylC**	**BrtJ**	**ColE**	**ColD**	**NocO**	**NocN**	**Mic**
**Strain/ Accession Number**	***Aliterella atlantica*/ WP_045054787.1**						
**Coverage**	94%	95%	93%	96%	95%	97%	98%
**E-value**	3,00E-147	4,00E-67	2,00E-153	7,00E-176	8,00E-124	0.0	7,00E-148
**Percent identity**	47.33%	41.70%	49.32%	51.72%	43.57%	69.52%	46.96%
**Strain/ Accession Number**	***Chroococcidiopsis sp. TS-821*/WP_104546385.1**						
**Coverage**	98%	99%	93%	99%	95%	99%	94%
**E-value**	4,00E-155	9,00E-68	8,00E-159	7,00E-175	1,00E-121	0.0	6,00E-150
**Percent identity**	48.65%	42.98%	51.93%	50.85%	43.15%	68.66%	49.22%
**Strain/ Accession Number**	***Gloeobacter violaceus SpSt-379*/HGZ84776.1**						
**Coverage**	94%	94%	95%	95%	100%	94%	95%
**E-value**	1,00E-57	1E-109	5,00E-92	1,00E-98	3,00E-87	3,00E-100	5,00E-62
**Percent identity**	42.79%	43.98%	36.00%	37.64%	34.39%	37.16%	46.73%

***Calothrix brevissima* NIES-22 DNA**

AP018207.1

17.5 kb

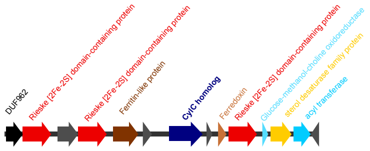

***Tolypothrix tenuis* PCC 7101**

AP018248.1

26.2 kb

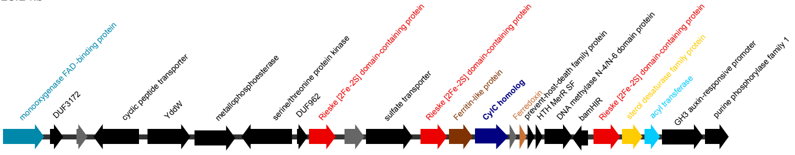

***Nostoc punctiforme* PCC 73102**

CP001037.1

Cluster 2

20.6 kb

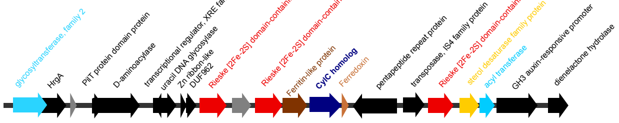

***Aulosira laxa* NIES-50**

AP018307.1

26.2 kb

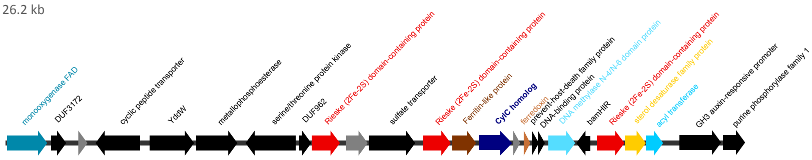

***Calothrix* sp. NIES-4071**

AP018255.1

Cluster 1

23.3 kb

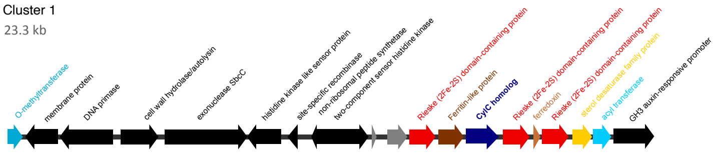

***Calothrix* sp. NIES-4105**

AP018290.1

Cluster 1

23.3 kb

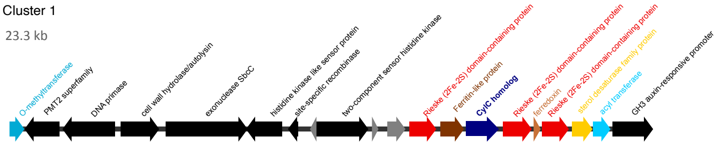

***Calothrix parasitica* NIES-267**

AP018227.1

12.2 kb

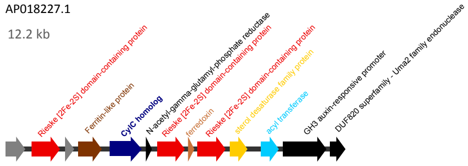

Calothrix **sp. NIES-2100**
AP018178.1
22.9 kb

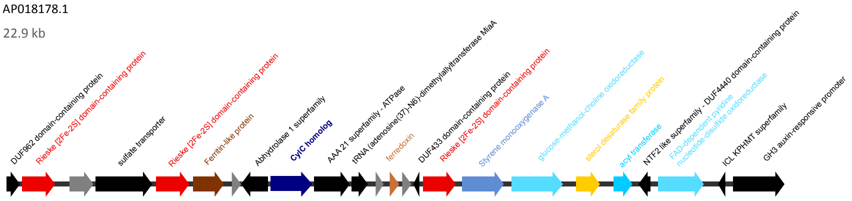

Geitlerinema **sp. PCC 7407**
CP003591.1
17.3 kb

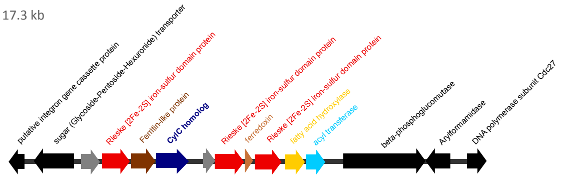

Nostoc **sp. CENA543**
CP023278.1
21.0 kb

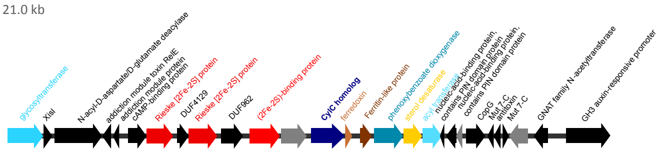

Cyanobacterium aponinum **PCC 10605**
CP003947.1
15.3 kb

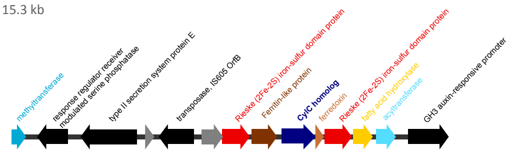

Calothrix **sp. NIES-2098 DNA**
AP018172.1
16.2 kb

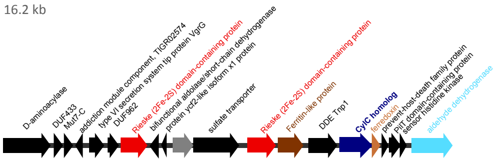

Nostoc **sp. 'Peltigera membranacea cyanobiont' N6**
CP026681.1
31.0 kb

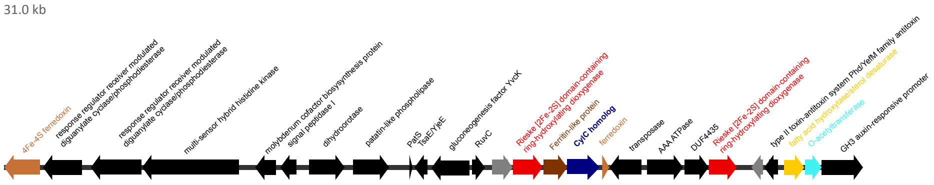

Nostoc carneum **NIES-2107**
AP018180.1
9.9 kb

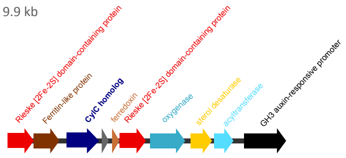

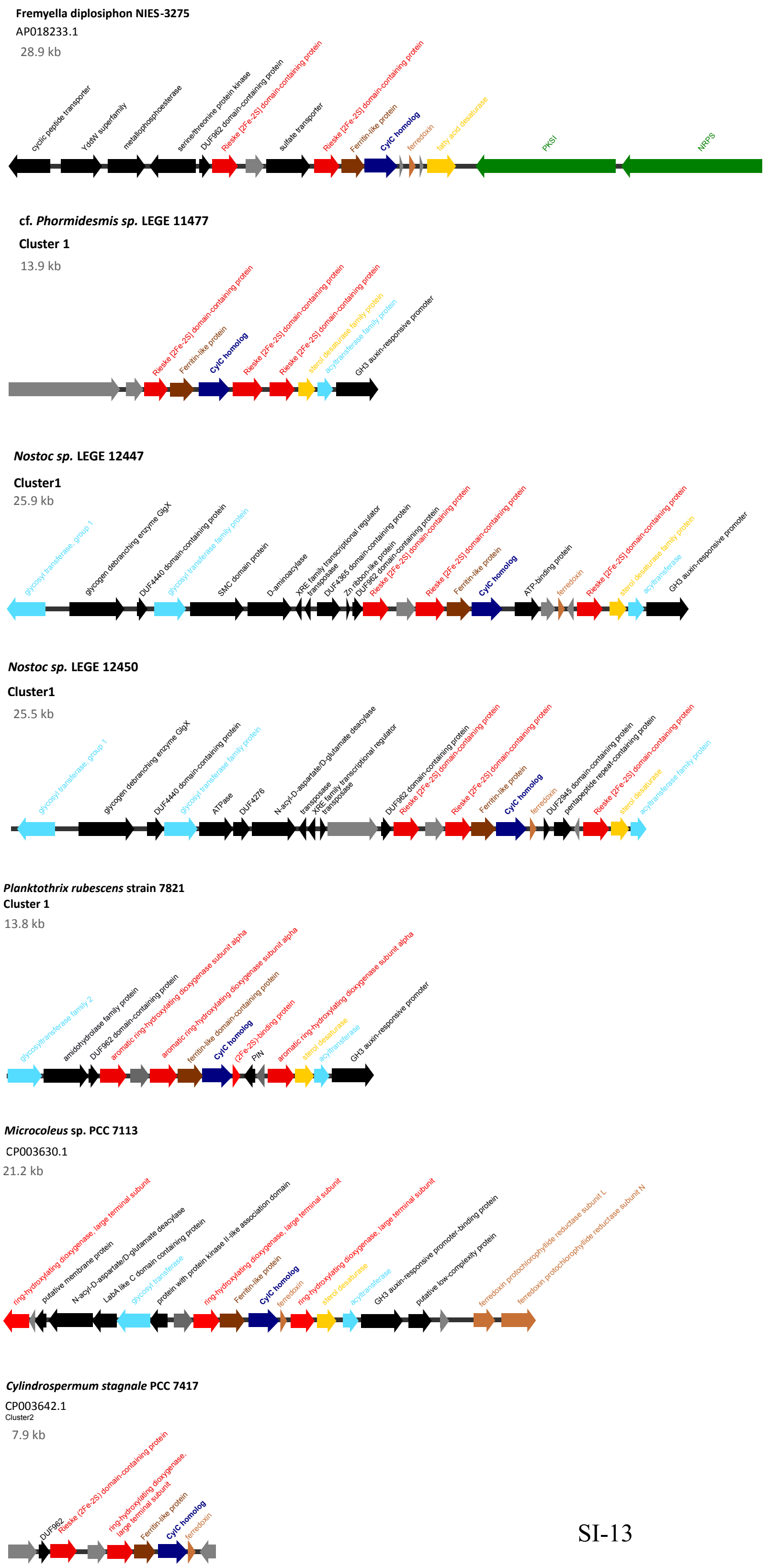
Fremyella diplosiphon NIES-3275
AP018233.1
28.9 kb
PKSI
NRPS
cf. Phormidesmis sp. LEGE 11477
Cluster 1
13.9 kb
Nostoc sp. LEGE 12447
Cluster1
25.9 kb
Nostoc sp. LEGE 12450
Cluster1
25.5 kb
Planktothrix rubescens strain 7821
Cluster 1
13.8 kb
Microcoleus sp. PCC 7113
CP003630.1
21.2 kb
Cylindrospermum stagnale PCC 7417
CP003642.1
Cluster2
7.9 kb

***Oscillatoria acuminata* PCC 6304**
CP003607.1
21.8 kb

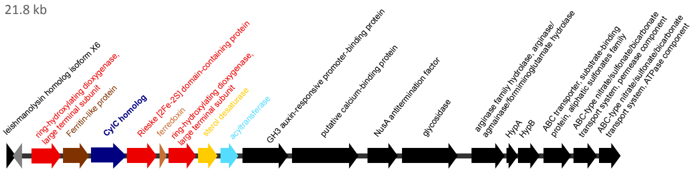

***Rivularia* sp. PCC 7116**
CP003549.1
11.4 kb

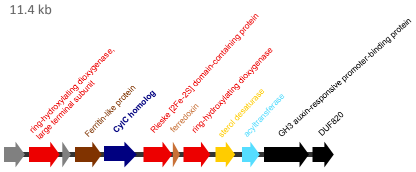

***Crinalium epipsammum* PCC 9333**
CP003620.1
9.3 kb

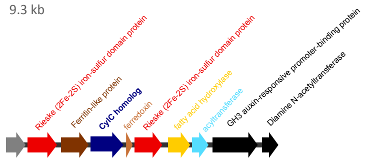

***Calothrix* sp. 336/3**
CP011382.1
12.8 kb

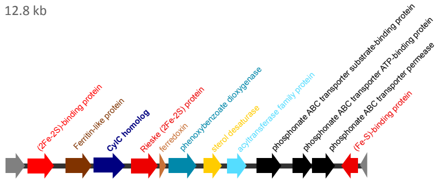

***Nostoc* sp. 'Lobaria pulmonaria (5183) cyanobiont' strain 5183**
CP026692.1
27.3 kb

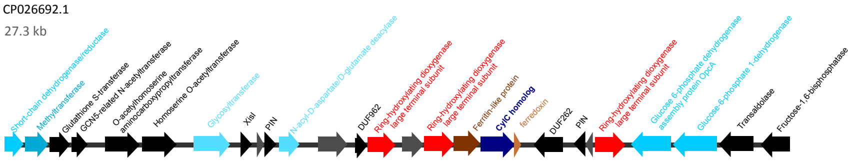

***Synechocystis* sp. LEGE 06083**
Cluster 1
10.2 kb

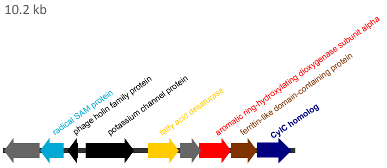

***Leptolyngbya saxicola* LEGE 07170**

10.3 kb

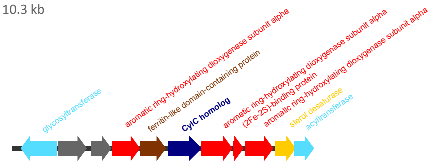

Tolypothrix **sp. LEGE 11397**

26.8 kb

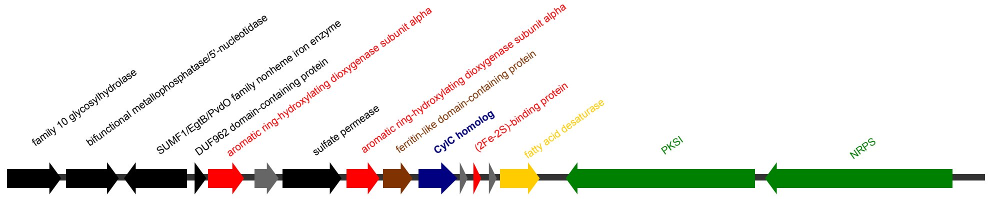

Leptolyngbya cf. ectocarpi **LEGE 11479**

15.9 kb

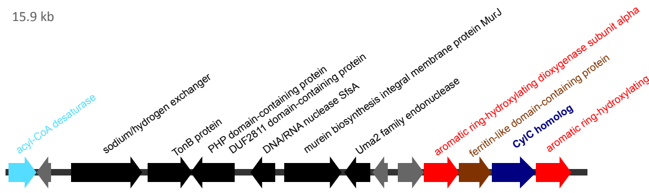

unidentified filamentous cyanobacterium LEGE 11480

16.5 kb

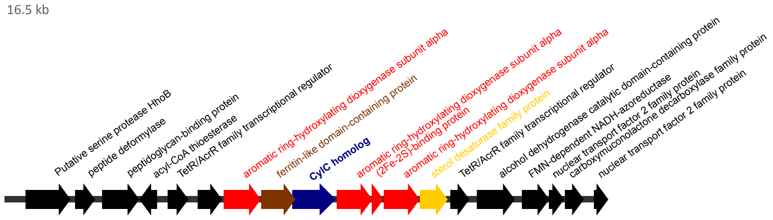

Planktothrix agardhii **NIVA CYA 15**

19.4 kb

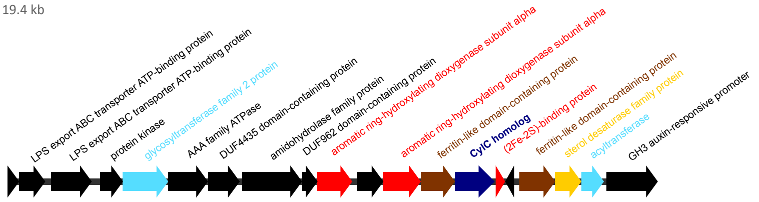

Planktothrix rubescens **NIVA CYA 406**
Cluster 1

30.3 kb

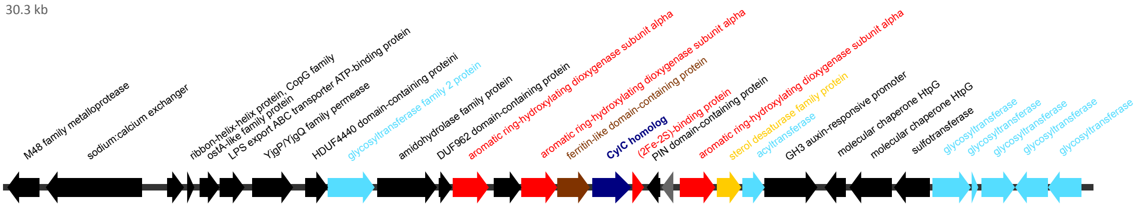

Planktothrix rubescens **NIVA CYA 407**

30.9 kb

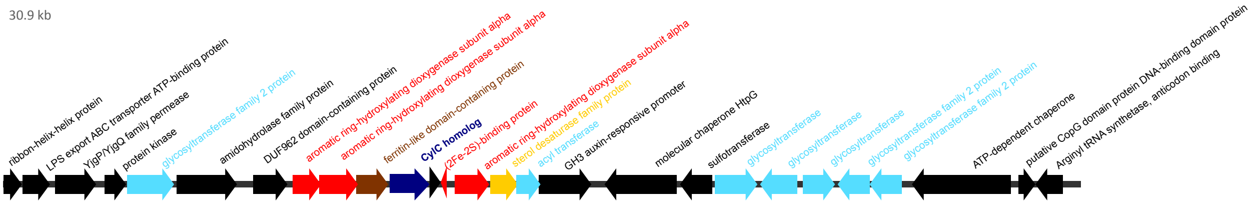

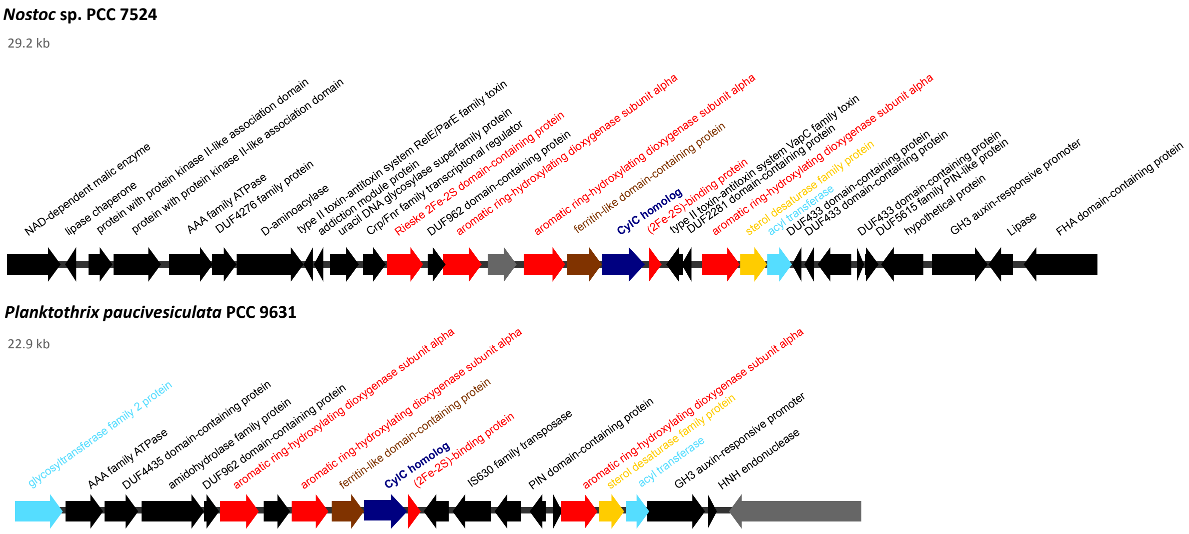

Figure S4. Rieske-containing biosynthetic gene clusters encoding CylC homolog(s).

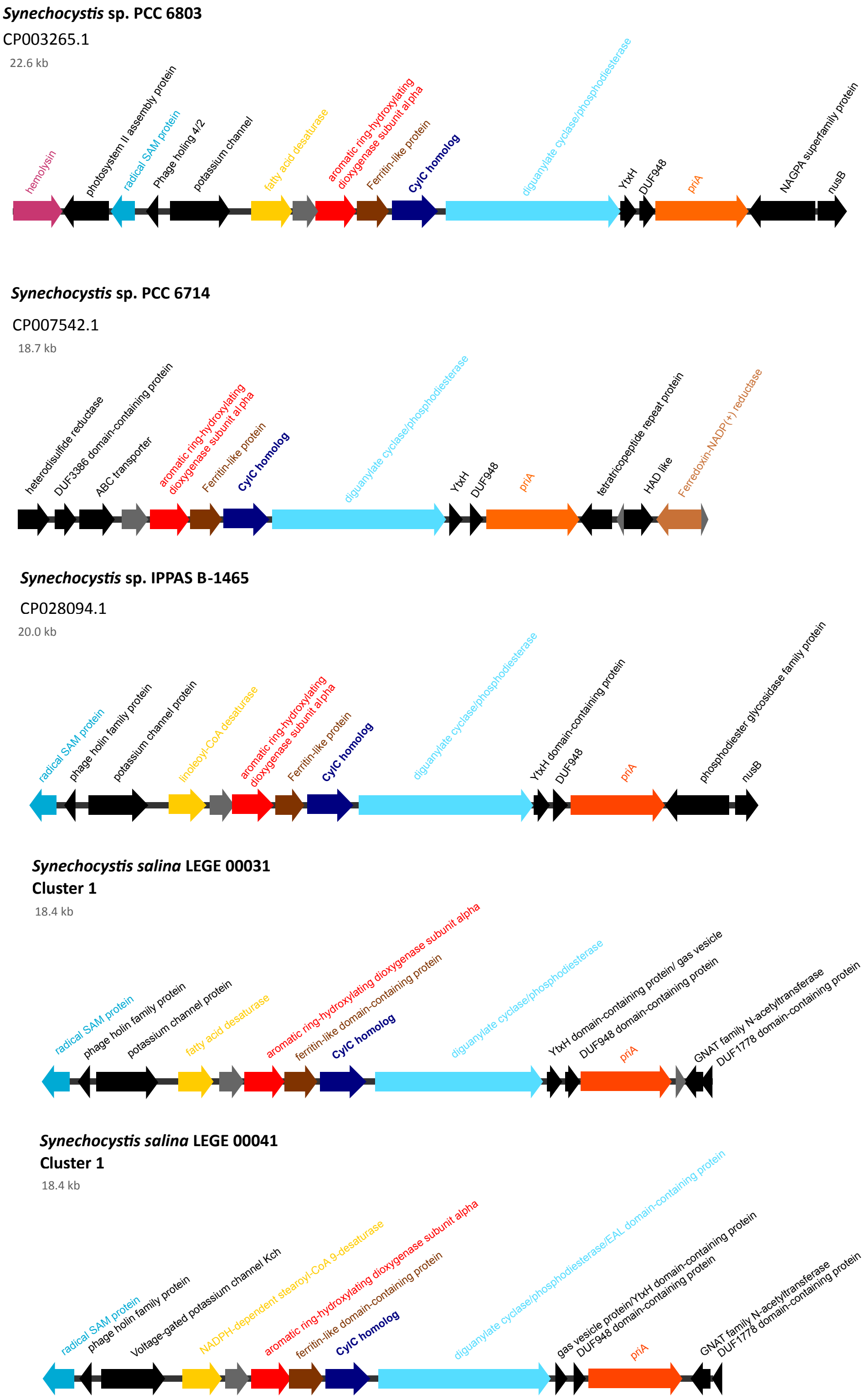

Figure S5. PriA-containing biosynthetic gene clusters encoding CylC homolog(s).

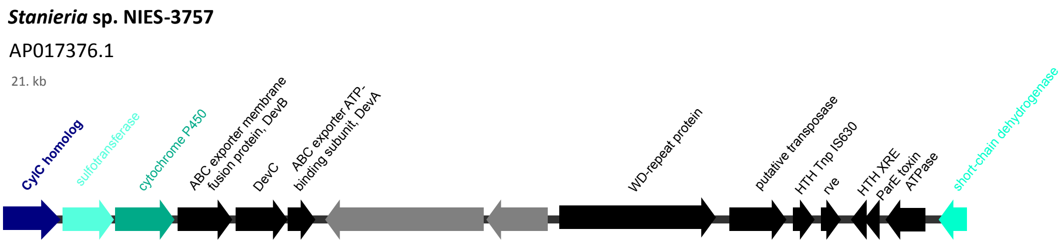

Figure S6. Cytochrome P450/sulfotransferase-containing biosynthetic gene cluster encoding a CylC homolog.

***Nostoc* sp. CCAP 1453/38**

20.9 kb

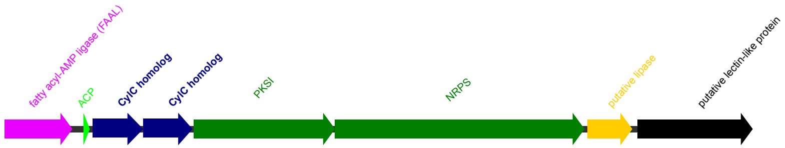

***Nodularia* sp. HBU26**

KY594676.1

23.2 kb

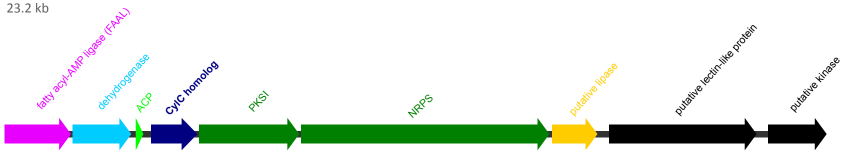

***Sphaerospermopsis* sp. LEGE00249**

chlorosphaerolactylates (cly)

30.9 kb

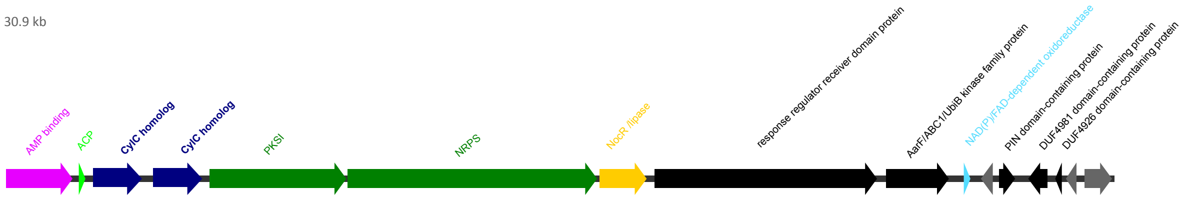

***Anabaena aphanizomenoides* LEGE 00250**

19.4 kb

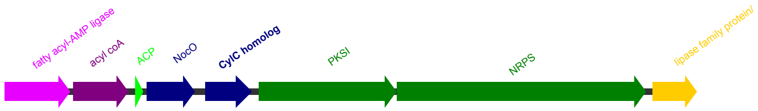

***Nodularia* sp. LEGE 06071**

24.2 kb

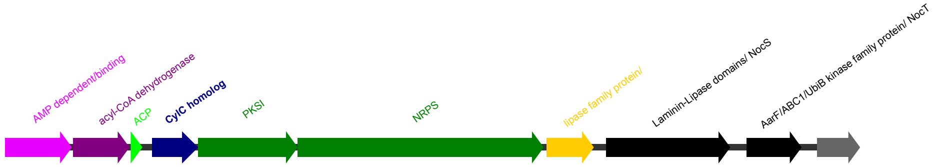

***Moorea bouillonii* PNG5-198**

KP715425.1 *columbamides (col)*

Cluster1

26.7 kb

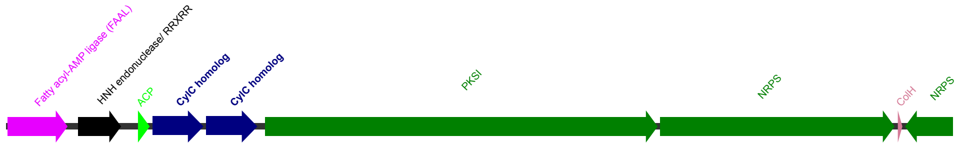

***Microcystis aeruginosa* 91341**

Cluster1

28.7 kb

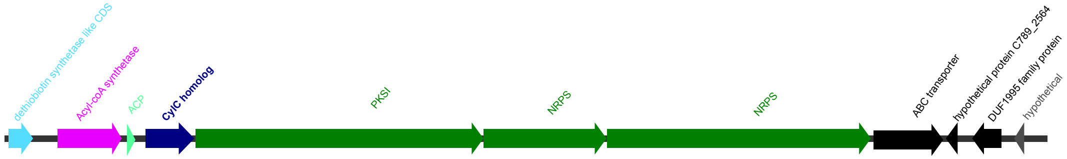

***Microcystis aeruginosa* PCC 9432**

microginin (mic)

26.7 kb

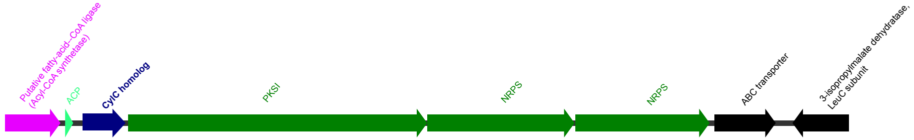

***Microcystis aeruginosa* FACHB-524**

49.0 kb

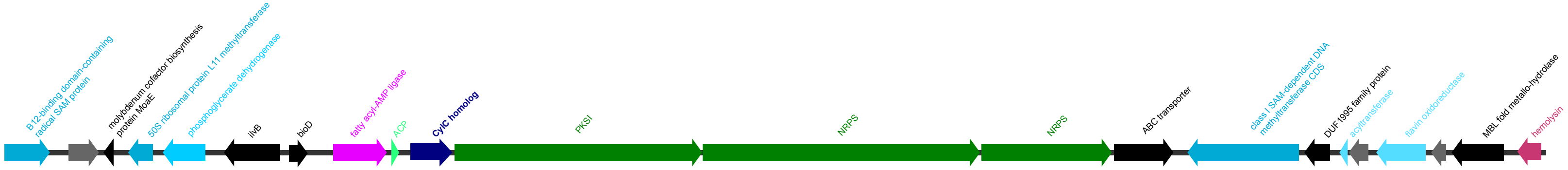

***Planktothrix rubescens* strain 7821**
Cluster 2

27.0 kb

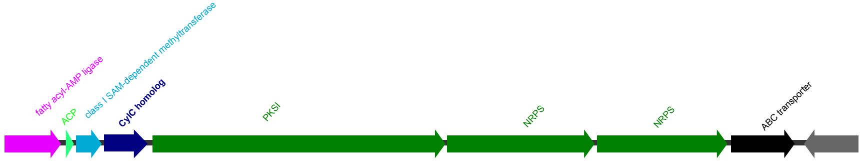

Microcystis aeruginosa **NIES 87**

28.0 kb

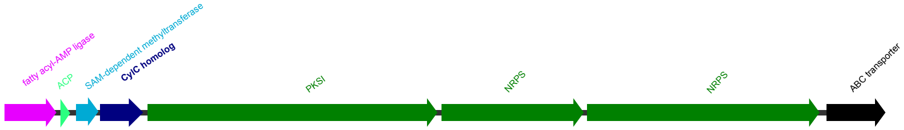

Microcystis aeruginosa **NIES 98**

31.7 kb

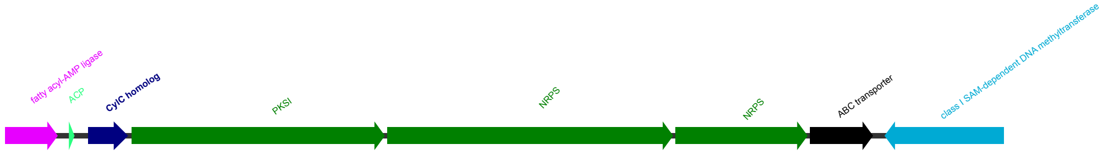

Planktothrix rubescens **NIVA CYA 406**
Cluster 2

25.1 kb

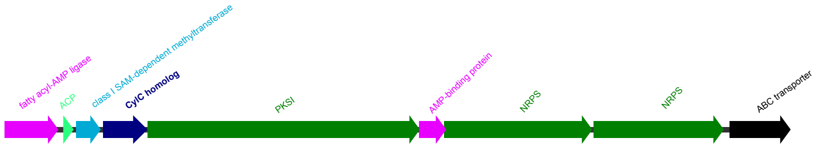

Microcystis aeruginosa **PCC 7005**

16.9 kb

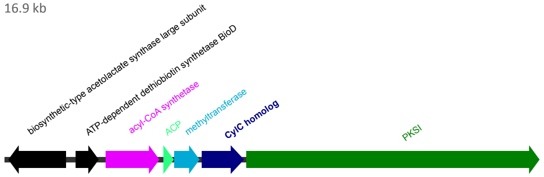

***Cylindrospermum stagnale* PCC 7417 plasmid**

46.1 kb

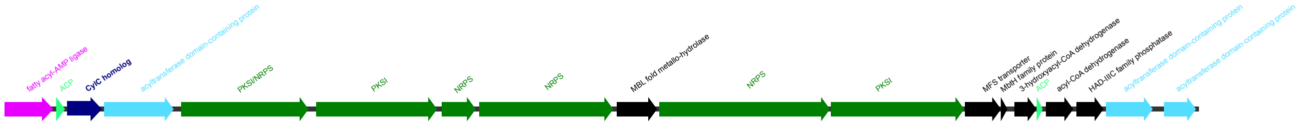

***Fischerella* sp. PCC 9431**
Cluster2

29.7 kb

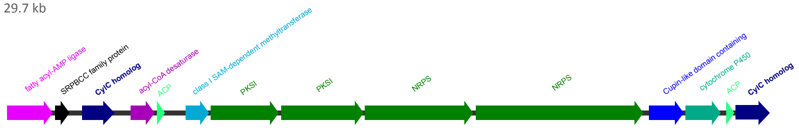

***Nostocales* cyanobacterium HT-58-2**

CP019636

35.3 kb

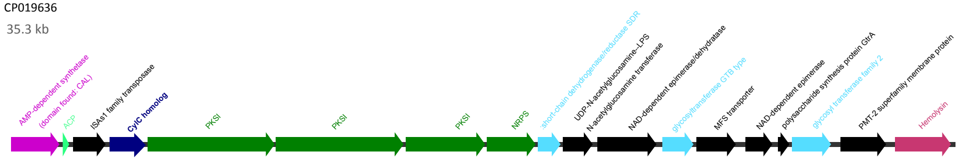

***Anabaena minutissima* UTEX B 1613**

MH325199.1

minutissamides (puwainaphycin-like, puw)

62.2 kb

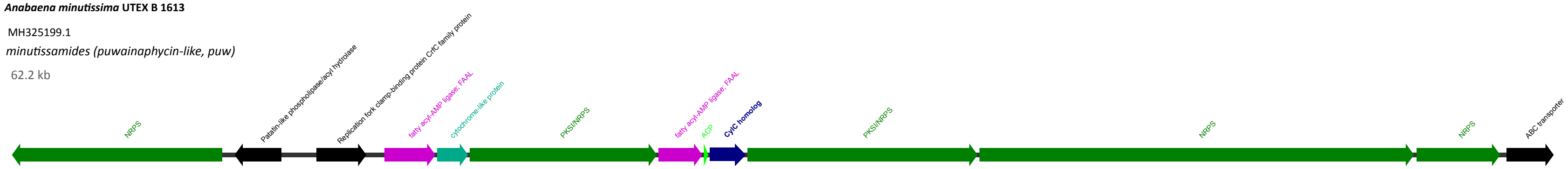

***Pleurocapsa* sp. PCC 7327**

CP003590.1

44.7 kb

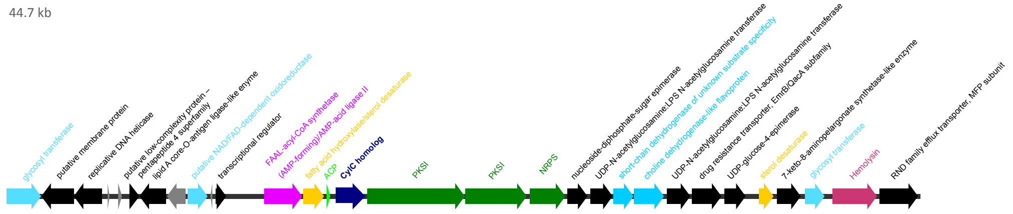

***Desmonostoc muscorum* LEGE 12446**

Cluster 1

37.2 kb

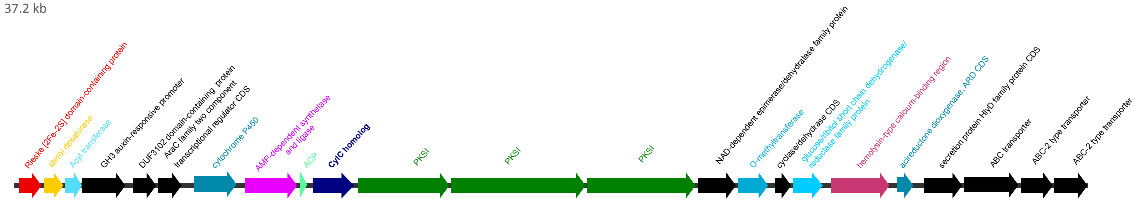

***Nostoc commune* HK-02**

AP018326.1

27.5 kb

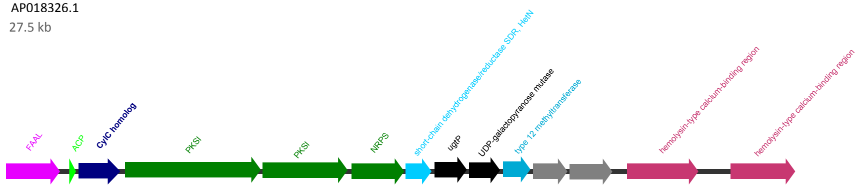

Nostoc punctiforme PCC 73102

CP001037.1

Cluster1

43.6 kb

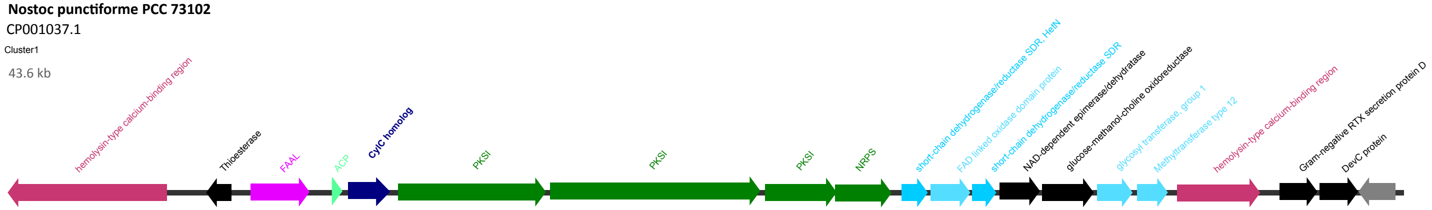

Nostoc sp. **LEGE 12447**
Cluster 3

26.9 kb

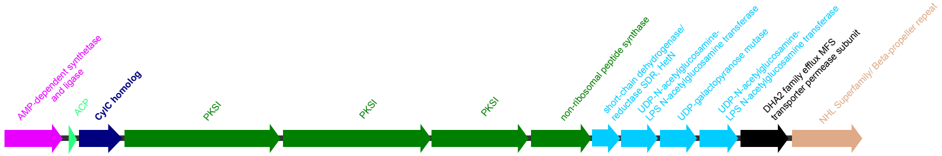

Nostoc **sp. NIES-4103**

AP018288.1

Cluster 2

37.3 kb

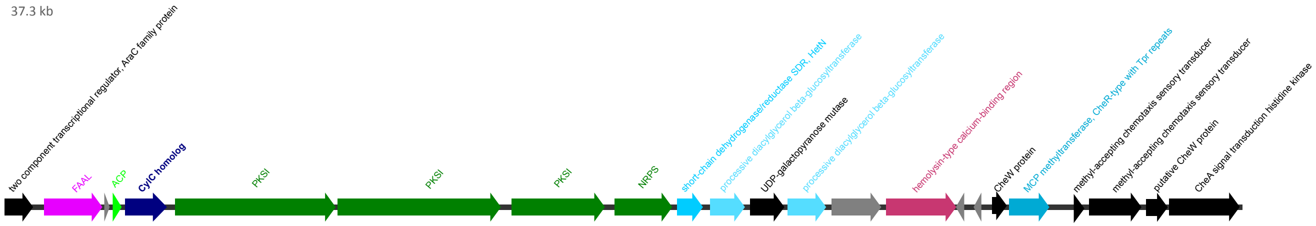

Cyanothece **sp. PCC 7822**

CP002199.1

41.1 kb

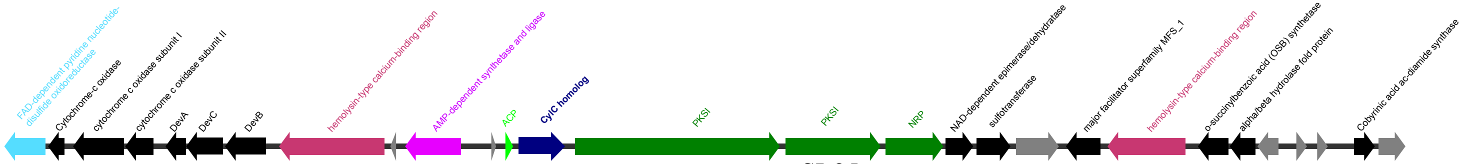

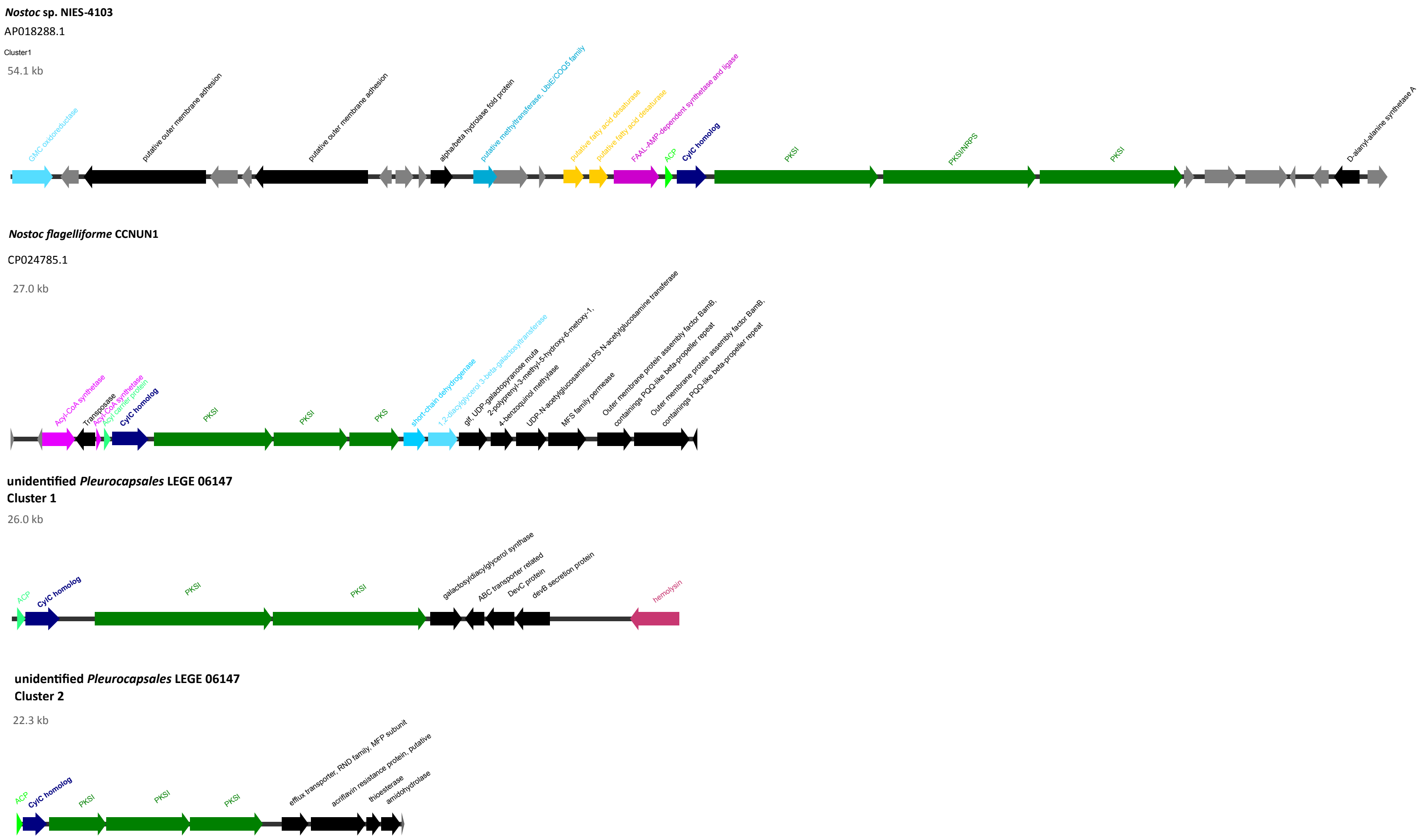

Figure S7. Type I PKS (chlorosphaerolactylate/columbamide/microginin/puwainaphycin-like) biosynthetic gene clusters encoding CylC homolog(s).

***Synechocystis salina* LEGE 06155**

Bartolosides

KR059027.1

16.4 kb

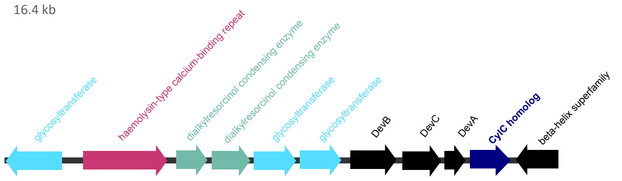

***Synechocystis salina* LEGE 06099**

Bartolosides

KX083339.1

14.4 kb

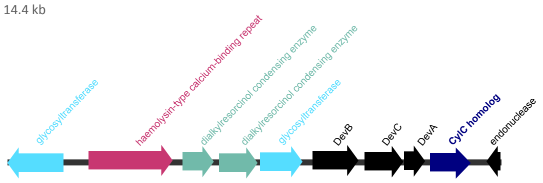

***Synechocystis salina* LEGE 00041**
Cluster 2

14.7 kb

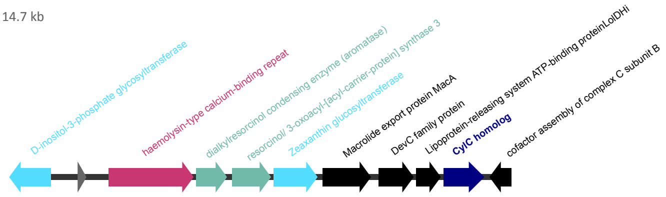

***Fischerella* sp. NIES-4106**

AP018298.1

32.9 kb

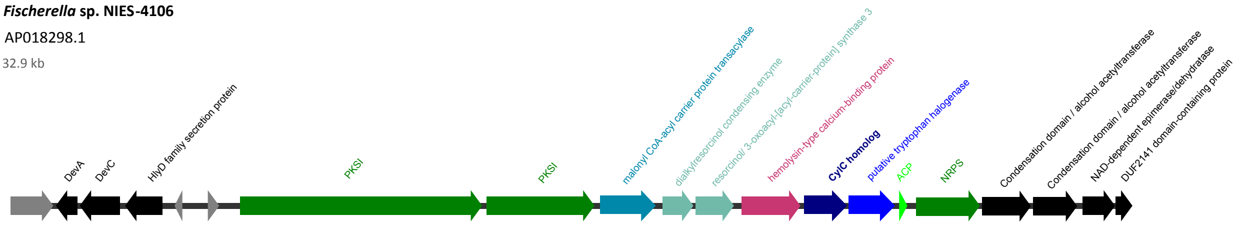

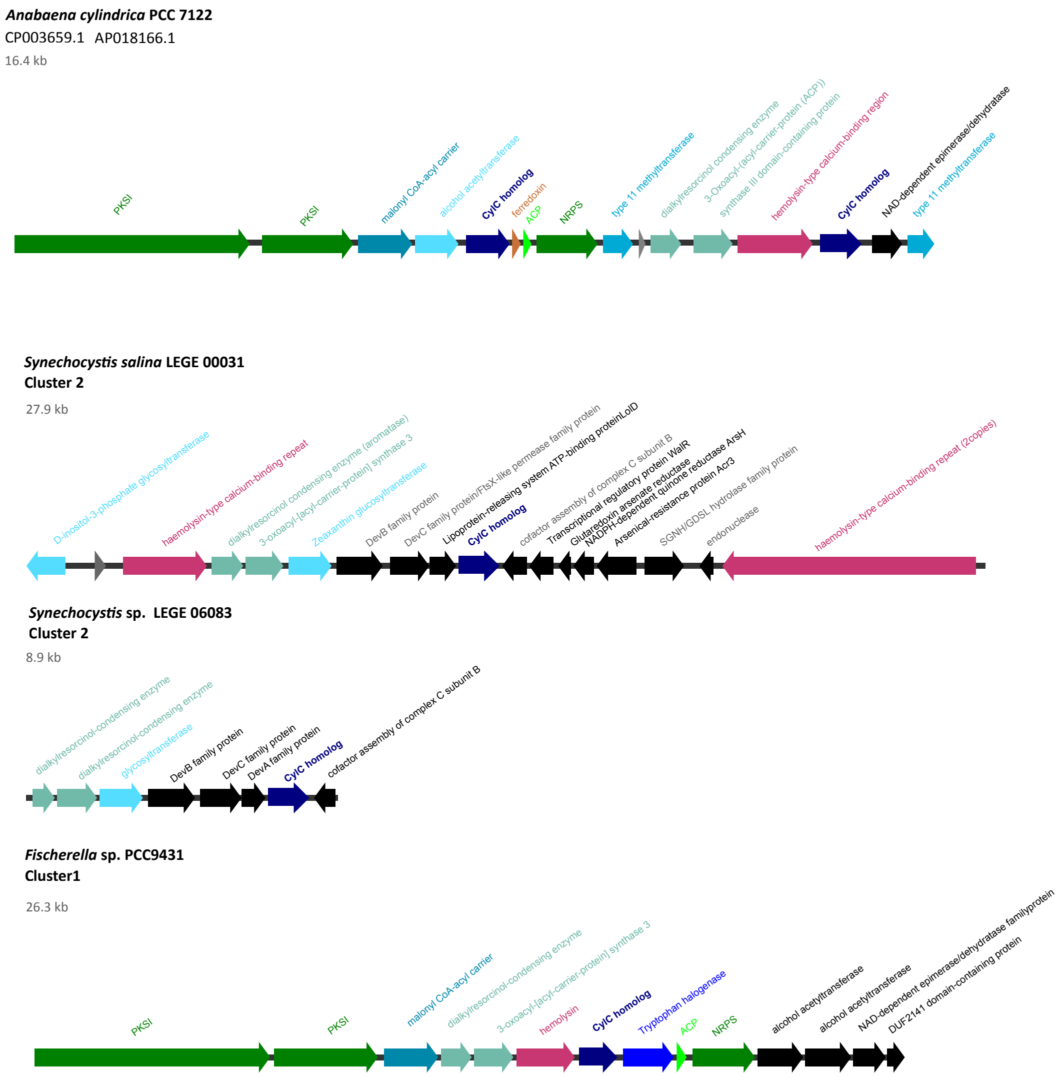

Figure S8. Dialkylresorcinol biosynthetic gene clusters encoding CylC homolog(s).

***Cylindrospermum licheniforme* UTEX B 2014** Cylindrocyclophane

KX682397.1

30.2 kb

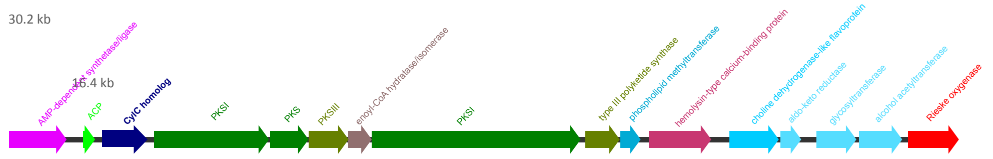

***Cylindrospermum stagnale* PCC 7417**

CP003642.1

Cluster1

36.4 kb

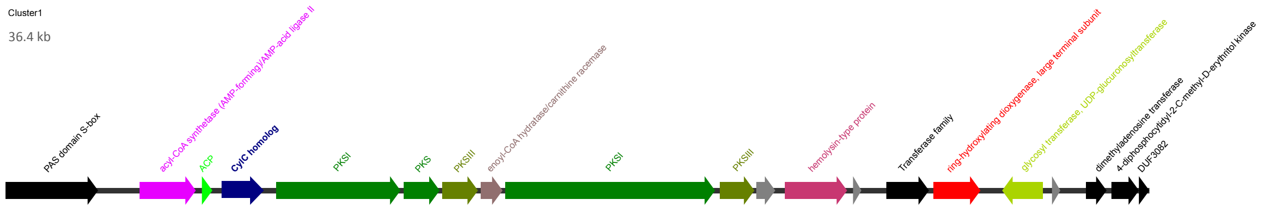

***Nostoc* sp. UIC 10110** Merocyclophane

KY379971.1

23.0 kb

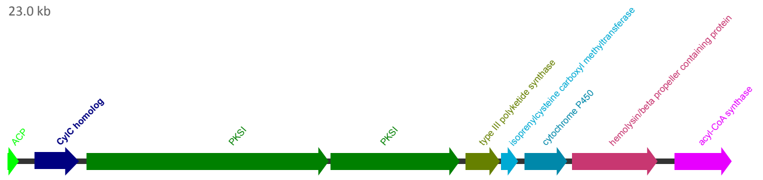

***Nostoc* sp. CAVN2** Carbamidocyclophane

KT826756.1

28.0 kb

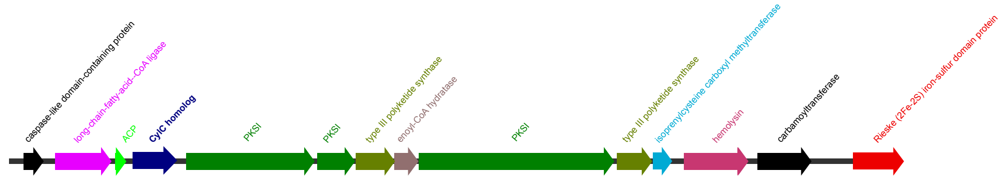

Cylindrospermum **sp. NIES-4074**

AP018269.1

24.0 kb

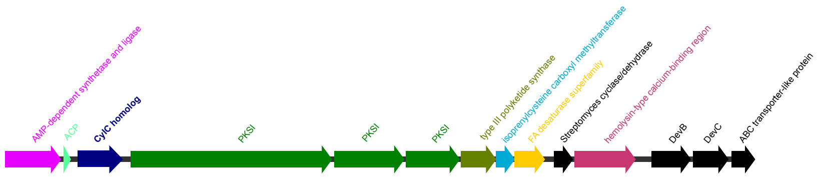

Calothrix **sp. NIES-4071**

AP018255.1

Cluster2

29.0 kb

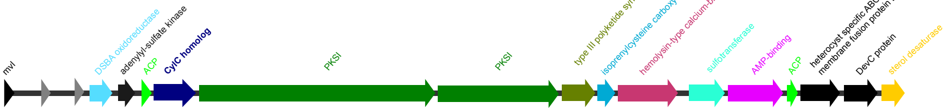

Calothrix **sp. NIES-4105**

AP018290.1

Cluster2

29.0 kb

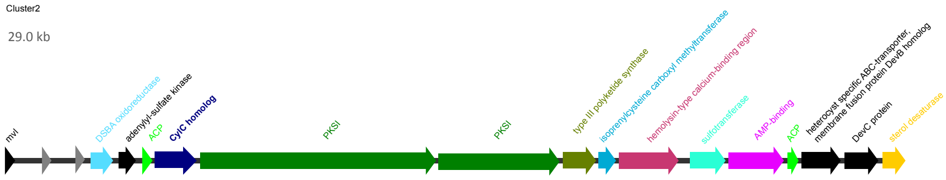

unidentified *Pleurocapsales* LEGE 10410

Cluster 1

30.0 kb

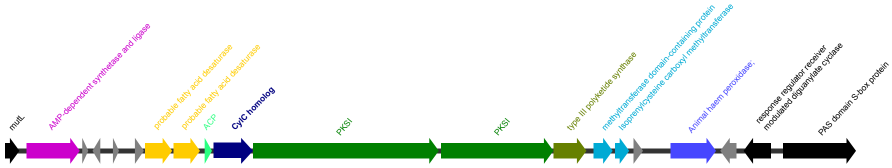

Hyella patelloides **LEGE 07179**

23.0 kb

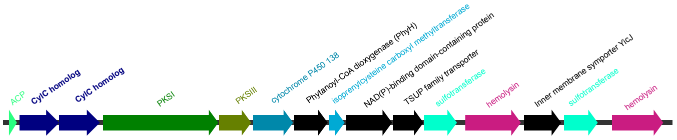

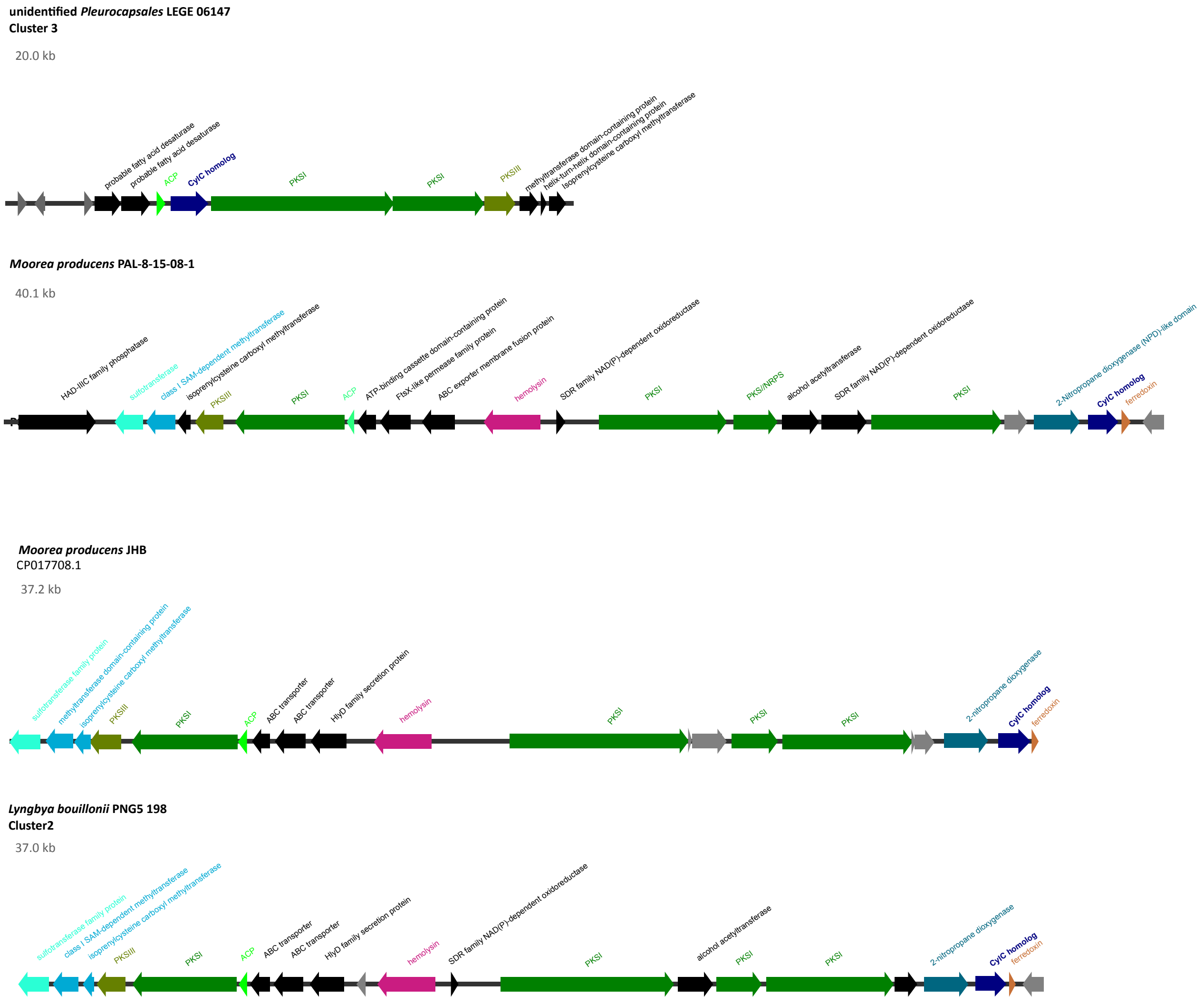

Figure S9. Type III PKS biosynthetic gene clusters encoding CylC homolog(s).

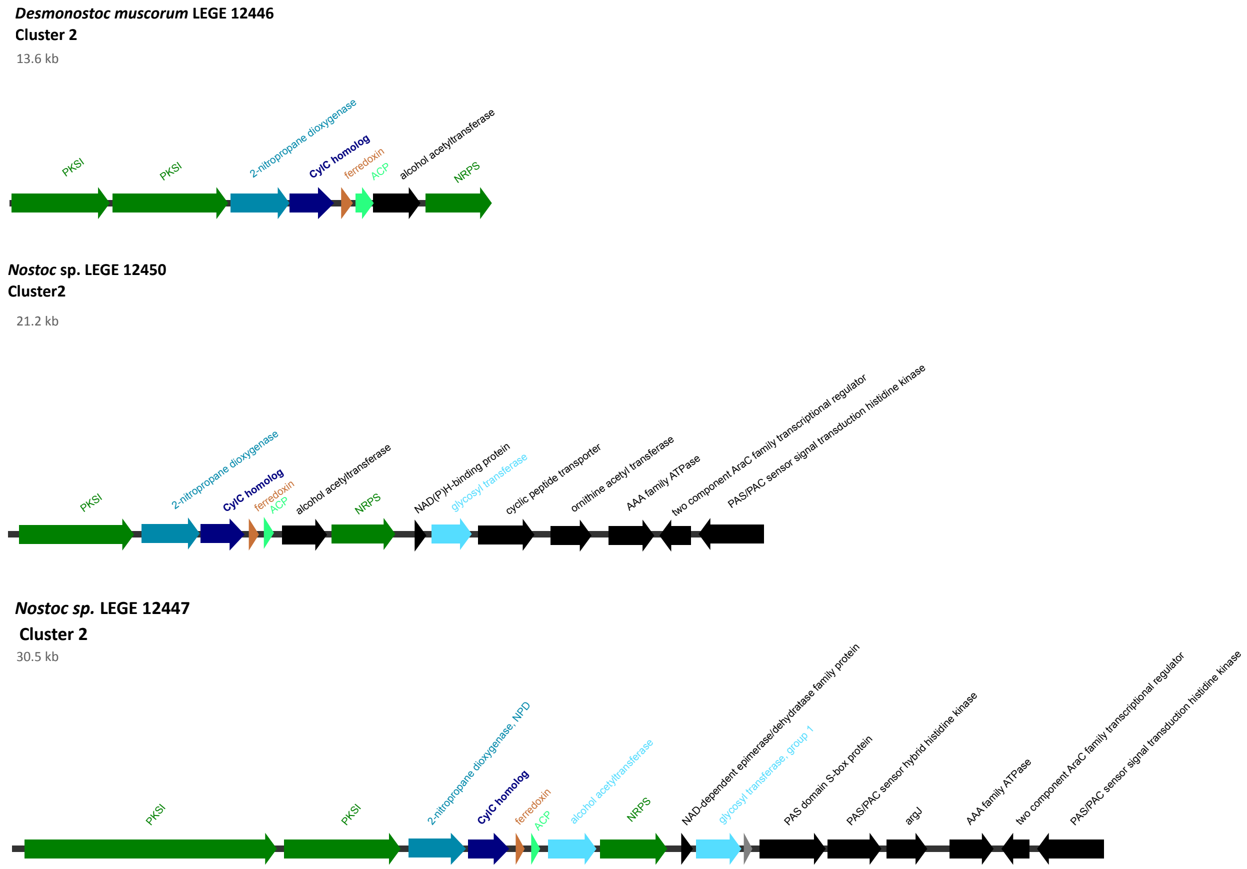

Figure S10. Nitronate monooxygenase-containing biosynthetic gene clusters encoding a CylC homolog.

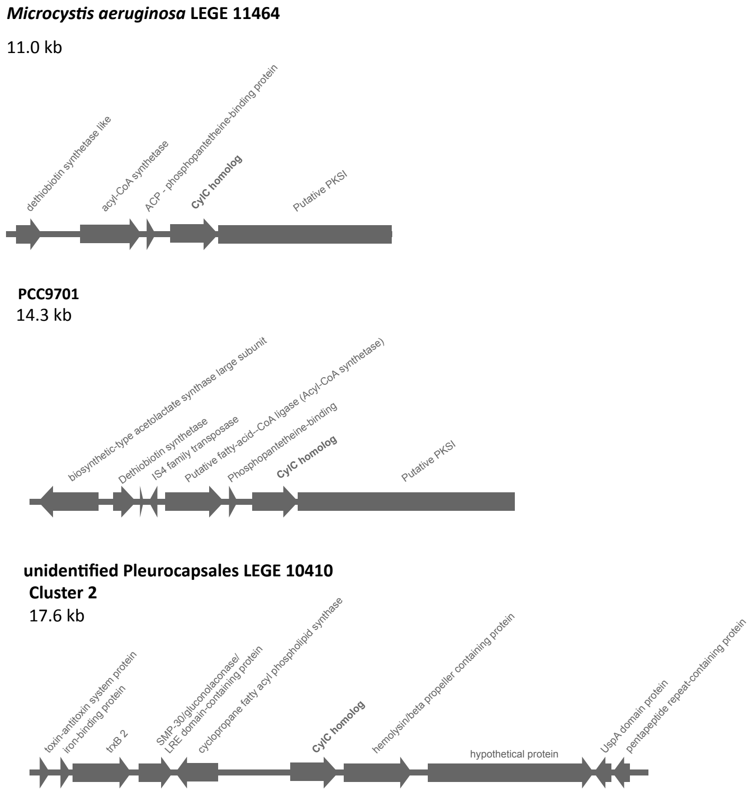

Figure S11. Unclassified (likely incomplete) biosynthetic gene clusters encoding a CylC homolog.

Table S4. BLAST search of Rieske-containing BGCs genes from *Calothrix brevissima* NIES 22 against *Synechocystis* sp. PCC 6803.

Gene	Annotation	PCC 6803 ortholog	E-value	Identity	Query Coverage	Bit-Score
**NIES22_66770**	DUF962 domain-containing protein	none				
**NIES22_66780**	Rieske aromatic ring-hydroxylating dioxygenase	sll1849	8.68E-97	68.6	61.63	311.227
**NIES22_66790**	hypothetical	sll0263	2.85E-67	46.4	84.92	222.246
**NIES22_66800**	Rieske aromatic ring-hydroxylating dioxygenase	sll0264	5.74E-137	60.2	97.01	426.402
**NIES22_66810**	ferritin-like diiron protein	sll0265	8.33E-74	43.3	98.67	243.817
**NIES22_66820**	hypothetical	slr0326	1.31E-17	67.1	78.65	72.789
**NIES22_66830**	CylC-like dimetal-carboxylate halogenase	sll0266	5.63E-158	60.8	94.37	489.574
**NIES22_66840**	hypothetical	none				
**NIES22_66850**	ferredoxin	ssl2559	1.79E-28	53.4	93.62	107.071
**NIES22_66860**	Rieske aromatic ring-hydroxylating dioxygenase	sll1297	3.15E-147	61.5	100	456.447
**NIES22_66880**	sterol desaturase or sphingolipid hydroxylase	sll1510	1.88E-59	45.9	99.59	199.904
**NIES22_66890**	acyl transferase	sll1511	3.08E-56	51	76.12	188.734

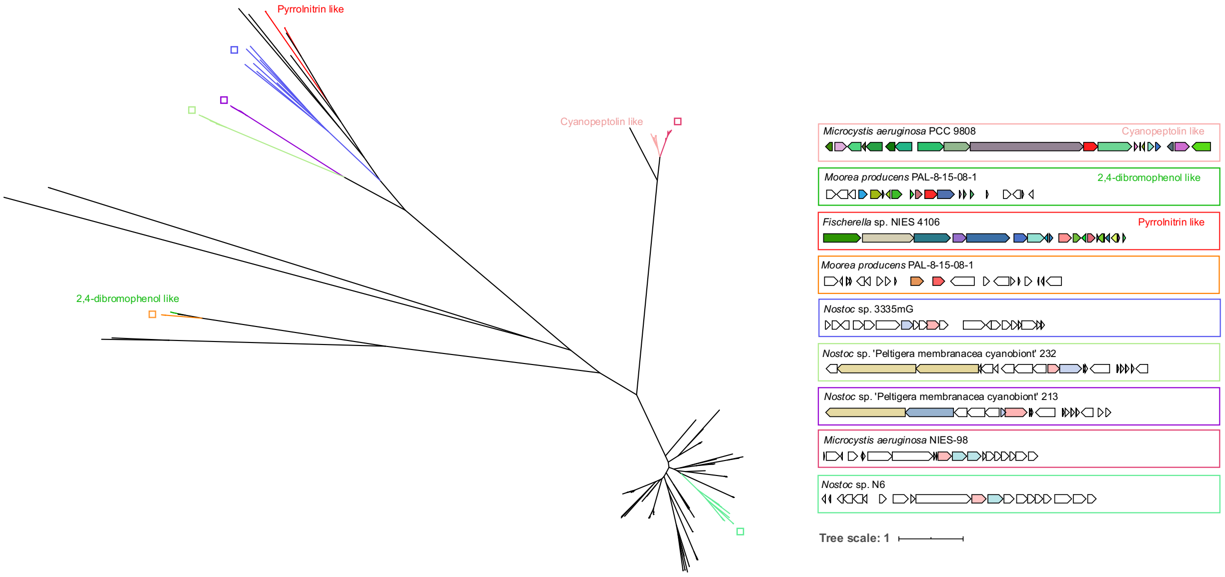

Figure S12. Phylogenetic tree of FAD-dependent halogenases based on CORASON outputs with illustrative BGC architectures.

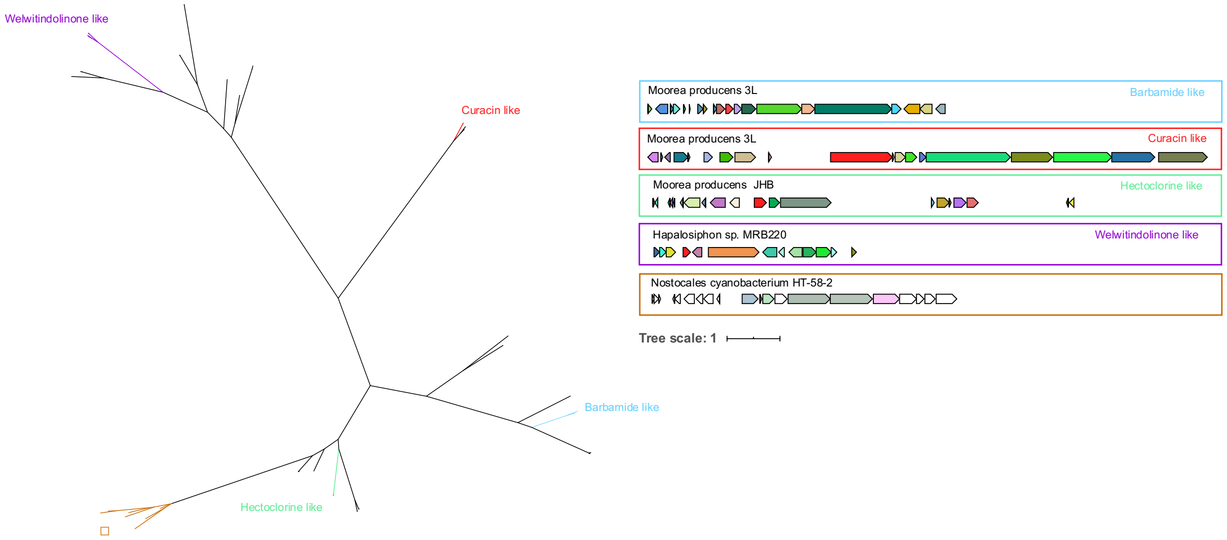

Figure S13. Phylogenetic tree of nonheme iron-dependent halogenases based on CORASON outputs with illustrative BGC architectures.

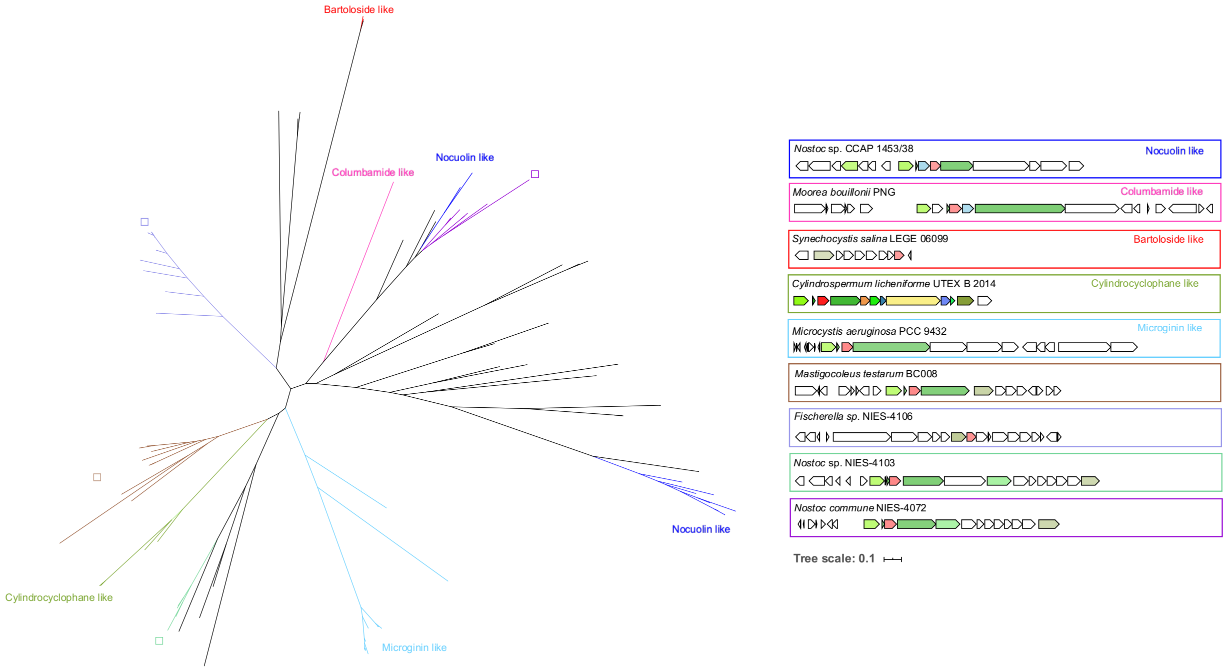

Figure S14. Phylogenetic tree of dimetal-carboxylate halogenases based on CORASON outputs with illustrative BGC architectures.
